# Ontogenetic Variation of Individual and Total Capsaicinoids in Malagueta Peppers (*Capsicum frutescens*) during Fruit Maturation

**DOI:** 10.3390/molecules22050736

**Published:** 2017-05-03

**Authors:** Oreto Fayos, Ana Carolina de Aguiar, Ana Jiménez-Cantizano, Marta Ferreiro-González, Ana Garcés-Claver, Julián Martínez, Cristina Mallor, Ana Ruiz-Rodríguez, Miguel Palma, Carmelo G. Barroso, Gerardo F. Barbero

**Affiliations:** 1 Agrifood Research Centre of Aragón (CITA), Montañana Avenue, 930, 50059 Zaragoza, Spain; ofayos@cita-aragon.es (O.F.); agarces@cita-aragon.es (A.G.-C.); cmallor@aragon.es (C.M.); 2 Department of Food Engineering, College of Food Engineering, University of Campinas, DEA/FEA/UNICAMP, Campinas 13083-862, SP, Brazil; aguiarea@gmail.com (A.C.d.A.); julian@fea.unicamp.br (J.M.); 3 Department of Chemical Engineering and Food Technology, Faculty of Sciences, University of Cádiz, Agrifood Campus of International Excellence (CeiA3), IVAGRO, P.O. Box 40, 11510 Puerto Real, Cádiz, Spain; ana.jimenezcantizano@uca.es; 4 Department of Analytical Chemistry, Faculty of Sciences, University of Cádiz, Agrifood Campus of International Excellence (CeiA3), IVAGRO, P.O. Box 40, 11510 Puerto Real, Cádiz, Spain; marta.ferreiro@uca.es (M.F.-G.); ana.ruiz@uca.es (A.R.-R.); miguel.palma@uca.es (M.P.); carmelo.garcia@uca.es (C.G.B.)

**Keywords:** capsaicinoids, *Capsicum frutescens*, Malagueta pepper, ontogenetic variation, pepper ripening, UHPLC

## Abstract

The ontogenetic variation of total and individual capsaicinoids (nordihydrocapsaicin (n-DHC), capsaicin (C), dihydrocapsaicin (DHC), homocapsaicin (h-C) and homodihydrocapsaicin (h-DHC)) present in Malagueta pepper (*Capsicum frutescens*) during fruit ripening has been studied. Malagueta peppers were grown in a greenhouse under controlled temperature and humidity conditions. Capsaicinoids were extracted using ultrasound-assisted extraction (UAE) and the extracts were analyzed by ultra-performance liquid chromatography (UHPLC) with fluorescence detection. A significant increase in the total content of capsaicinoids was observed in the early days (between 12 and 33). Between day 33 and 40 there was a slight reduction in the total capsaicinoid content (3.3% decrease). C was the major capsaicinoid, followed by DHC, n-DHC, h-C and h-DHC. By considering the evolution of standardized values of the capsaicinoids it was verified that n-DHC, DHC and h-DHC (dihydrocapsaicin-like capsaicinoids) present a similar behavior pattern, while h-C and C (capsaicin-like capsaicinoids) show different evolution patterns.

## 1. Introduction

**Figure 1:**
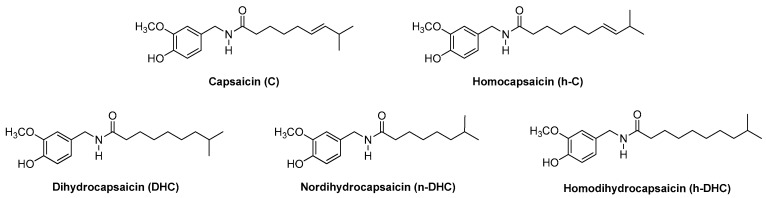
Chemical structures of the major capsaicinoids capsaicin, homocapsaicin, dihydrocapsaicin, nordihidrocapsaicin and homodihydrocapsaicin.

Hot peppers are widely used as a flavoring and a spice throughout the world due to their color, aroma and characteristic pungency [1,2]. Peppers are the only plants that are able to produce alkaloids belonging to the capsaicinoid family and these compounds are responsible for the characteristic pungency. Capsaicinoids are nonvolatile alkaloids that are acid amides of C_9_–C_11_ branched-chain fatty acids and vanillylamines [3]. Capsaicin (C) and dihydrocapsaicin (DHC) are the predominant molecules and they represent up to 90% of total capsaicinoids. Some other related compounds, such as nordihydrocapsaicin (n-DHC), homocapsaicin (h-C) and homodihydrocapsaicin (h-DHC), are also present in minor amounts [4,5] (Figure 1). In *Capsicum* plants these compounds are synthesized naturally in the placenta of the pepper fruit, where they accumulate in vesicles, by enzymatic condensation of vanillylamine and different-sized fatty acid chains that are elongated by a fatty acid synthase [6].

Capsaicinoids have attracted a great deal of attention in recent years due to their proven strong pharmacological properties. They can be used in pain relief, cancer prevention and weight reduction, and they also have gastrointestinal and cardiovascular benefits [7,8,9,10].

It has been demonstrated that capsaicinoids are synthesized and accumulated in the placenta of peppers [11,12]. In general, the results of studies carried out to elucidate the process of accumulation of capsaicinoids in *Capsicum* fruits over the maturation period indicate that these compounds begin to accumulate in the early stages of fruit development and then continue to accumulate until they reach a maximum, which is usually found after 40–60 days of ripening [13,14,15,16,17,18]. Beyond this point the trend reverses due to the degradation of capsaicinoids associated with the action of peroxidases, which are highly capable of degrading C and DHC [19,20]. In some studies there are certain varieties of peppers (Peter Pepper) in which this decrease in the total content of capsaicinoids was not observed over time [21]. Capsaicinoid accumulation and the activity of the corresponding biosynthetic enzymes are sensitive to environmental conditions such as the availability of water, the stage of fruit development [15,22,23], mineral nutrition [24,25] and any infections suffered by the pepper plants [26]. Furthermore, it is known that the capsaicinoid content of peppers can vary between different fruits within the same plant, even when they are harvested at the same time after flowering [27], and in peppers that have different node positions [28]. Something similar happens with various compounds in plants of the family *Molluginaceae* [29]. The most widely used technique for the analysis of capsaicinoids is reversed phase-high performance liquid chromatography (RP-HPLC) [4,30,31,32,33]. In recent years, reversed phase-ultra-performance liquid chromatography (RP-UHPLC) has become a more effective and more rapid alternative for the analysis of capsaicinoids in peppers [34,35,36,37].

*Capsicum* peppers have a high economic importance in Brazil since this country is a center of genetic diversity and one of the world’s largest pepper producers [38]. In 2005, *Capsicum* peppers were the second-most exported vegetable from Brazil, with an export volume of 9222 tons. Malagueta pepper (*Capsicum frutescens*) is one of the most popular domestic varieties that is cultivated in the Brazilian territory [together with Dedo-de-Moça (*Capsicum bacccatum* var. *pendulum*) and Murupi (*Capsicum chinense*) peppers] [39,40]. Malagueta pepper is also widely consumed in Portugal, Angola and Mozambique. It is a small, tapered green pepper of about 5 cm in length that turns red as it matures. Two sizes are seen in markets and these sometimes are given different names: the smaller ones are called “malaguetinha” in Brazil and “piri piri” in Portugal and Mozambique, and the larger ones are called “malagueta” in Brazil and Portugal. This pepper is used to season many regional dishes and sauces, including poultry dishes in Portugal [41,42,43].

The aim of the work described here was to evaluate the accumulation of individual and total capsaicinoids (n-DHC, C, DHC, h-C and h-DHC) during the ripening of Malagueta peppers. One of the most important parameters in commercial Malagueta peppers is the capsaicinoid content. In this way, farmers would be able to collect Malagueta peppers according to their capsaicinoid content.

## 2. Results and Discussion

### 2.1. Evolution of the Total Capsaicinoid Content in Malagueta pepper

Malagueta pepper plants began to produce peppers in the second week of July. The peppers were harvested on September 30 (11 weeks later). The monitoring was carried out during maturation starting 12 days after the appearance of the first pepper and was continued to a state of over-ripeness (S-10, Table 1). In the state of over-ripeness, peppers showed water loss (dehydration) and a very intense red coloration. The visual states of the peppers at harvest are described in Table 1.

**Figure 2:**
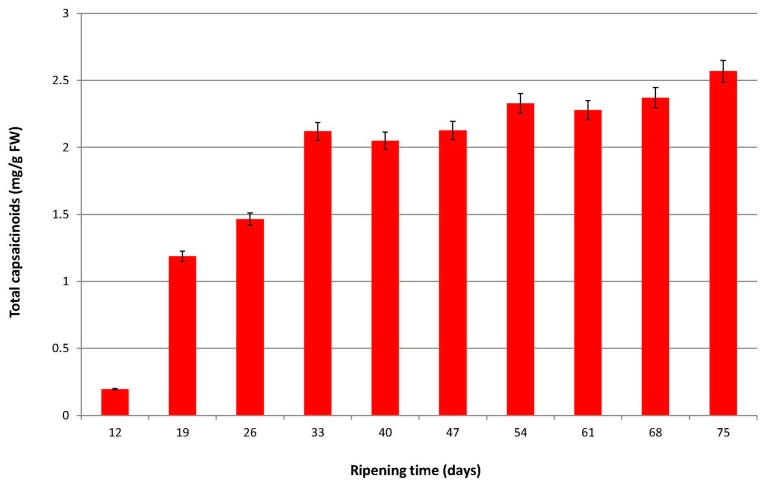
Total capsaicinoids (mg g^−1^ FW) in Malagueta pepper during fruit ripening (*n* = 3).

It can be seen from Figure 2 that the total capsaicinoid content in Malagueta pepper increased from the first point of harvest at 12 days (0.829 mg capsaicinoid g^−1^ FW) up to 33 days, where it reached a concentration of 2.119 mg capsaicinoid g^−1^ FW. Between days 33 and 40 there was a slight reduction in the total capsaicinoid content (3.3% decrease). From day 40 there was a slight increase in the total capsaicinoid content until day 54 (2.328 mg capsaicinoid g^−1^ FW). From day 54 a slight reduction in the total capsaicinoid content was again observed until day 61 (2.2% decrease). Finally, a further increase was observed in the total content of capsaicinoids due to the loss of water. In a global context, the total capsaicinoids content in Malagueta pepper rises from the first point of harvest at 12 days until 75 days, at which point there is a concentration of 2.568 mg capsaicinoid g^−1^ FW, which corresponds to an increase of over 300% compared to the initial capsaicinoid content.

Generally, studies on the accumulation of capsaicinoids in *Capsicum* fruits have shown a concentration increase in these compounds during the early stages of fruit development, and this is maintained during ripening until a maximum value is reached, usually between days 40 and 60 [13,14,15,16,18]. After this time there is a reverse in the trend and a marked reduction in the capsaicinoid content is observed. This change is associated with the presence of peroxidases that are capable of degrading C and DHC, as evidenced by the results of in vitro experiments performed by Bernal et al. [19,20].

In the study reported here, two perceptible decreases were observed in the amount of capsaicinoids during the maturation period: a decrease of approximately 3.3% was observed between days 33 and 40 and a reduction of 2.2% was verified between days 54 and 61. We consider that this second decrease in the capsaicinoid content could be due to both the action of the peroxidases and the reduced synthesis of capsaicinoids in the pepper due to the cultivation conditions in the greenhouse. The final increase in the total content of capsaicinoids is because of the loss of water by overripening of the peppers. As a consequence, it cannot be stated that a marked reduction in the levels of capsaicinoids occurs in the Malagueta pepper during the ripening time, a finding in contrast to that obtained in other accumulation studies available in the literature. Furthermore, the onset of the decrease in the total capsaicinoid content was observed at day 33 of ripening. The onset of this decrease is also more rapid when compared to those in the aforementioned studies [13,14,15,16,18]. In the actual study, in contrast to what is generally observed in the literature a significant increase in the content of capsaicinoids is observed in the early days (between 12 and 33), followed by two more modest increase to the end of the maturation process of the peppers. It is believed that this behavior may be due to the growing conditions in the greenhouse, where temperature, humidity, irrigation and fertilization were all controlled. A similar trend has been observed in Peter Pepper when it was grown under controlled conditions in a greenhouse [21]. According to these results it can be observed that in this variety of pepper a notable decrease in the total capsaicinoid content as happens with other varieties did not occur. Therefore, peppers could be collected (if so desired) from day 33, to harvest them with a high concentration of capsaicinoids, since from this moment on the total capsaicinoid concentration remains practically constant.

### 2.2. Evolution of the Individual Contents of Capsaicinoids in Malagueta pepper

Five capsaicinoids—n-DHC, C, DHC, h-C and h-DHC—were identified in Malagueta peppers at different ripening stages. The concentration of each individual capsaicinoid (expressed as mg capsaicinoid g^−1^ FW) in Malagueta pepper during fruit ripening is shown in Table 2. C was the major capsaicinoid, followed by DHC, n-DHC, h-C and h-DHC. The capsaicinoid profile is consistent with that reported by Aguiar et al. [44], with values of 1.02 mg g^−1^ FW for C and 0.496 mg g^−1^ FW for DHC from Malagueta obtained in a study of native peppers from Brazil. In this variety of pepper, capsaicin is the major capsaicinoid throughout maturation, unlike other pepper varieties, like Cayenne pepper, in which the major capsaicinoid switches between C and DHC during fruit ripening [13].

An overall increase in the concentration of all capsaicinoids can be observed with ripening (Table 2). The major capsaicinoids C and DHC increased by approximately 300%, whereas the minor capsaicinoids n-DHC, h-C and h-DHC increased by 430%, 390% and 470%, respectively. Regarding the decrease in total capsaicinoids that occurs between days 33 and 40 of fruit ripening, a decrease was observed with respect to individual capsaicinoids between days 33 and 40 for C and h-C (capsaicin-like capsaicinoids) and a decrease between days 33 and 47 for n-DHC, DHC and h-DHC (dihydrocapsaicin-like capsaicinoids).

**Figure 3:**
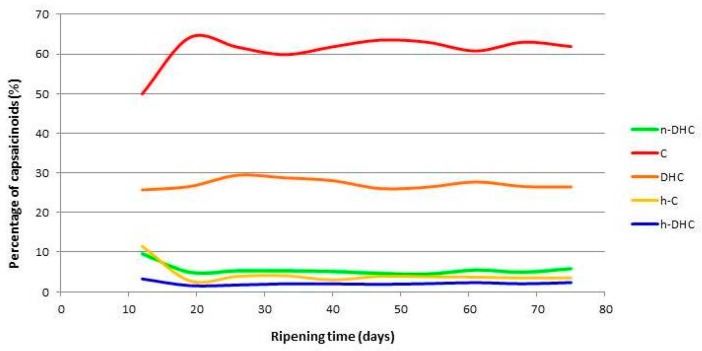
Percentages of individual capsaicinoids in Malagueta pepper during fruit ripening (*n* = 3).

The percentages of capsaicinoids found in Malagueta pepper are represented in Figure 3. A very slight variation can be observed between the different percentages of individual capsaicinoids over the ripening period. C and DHC were the major capsaicinoids and they correspond to around 90% of total capsaicinoids throughout the maturation period. This finding is in contrast to the behavior found in other varieties such as Cayenne pepper, in which the variability during fruit ripening is much more marked [13]. Capsaicin and dihydrocapsaicin have very similar biological and spicy properties. Capsaicin has 16,000,000 Scoville heat units (SHU) while dihydrocapsaicin 15,000,000 (SHU). There are only small differences between them. The other three capsaicinoids have lower pungency values, 9,100,000 SHU for n-DHC and 8,600,000 SHU for h-C and h-DHC [45,46,47]. In this case it is tested that the two main capsaicinoids in the Malagueta pepper are the ones that present greater pungent and biological properties.

The concentration of C ranged from 60% (33rd day) to 64% (19th day), whereas for DHC the lowest concentration of 26% was observed after 47 days and 29% after 26 days. Lower percentages were found for the minor capsaicinoids at the first harvest time (12 days) (4.21% for n-DHC, 2.76% for HC and 1.54% for h-DHC) and higher percentages were determined in the later period (75 days) (n-DHC, 5.83%; h-DHC, 2.33%), with the exception of h-C, which had the highest concentration at 33 days (4.08%).

### 2.3. Evolution of the Standardized Values of Capsaicinoids in Malagueta Pepper

**Figure 4:**
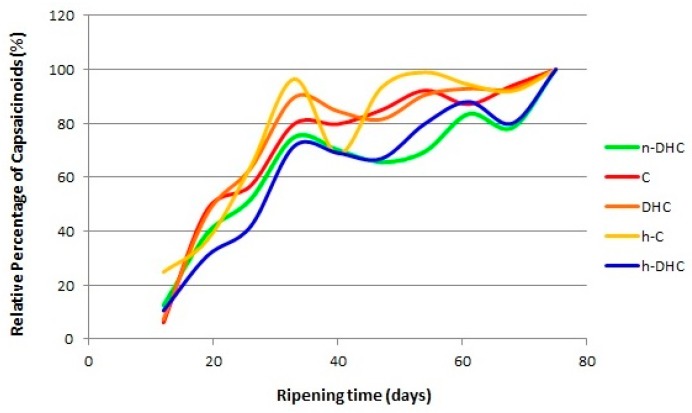
Relative percentages of individual capsaicinoids (standardized values) in Malagueta pepper during fruit ripening (*n* = 3).

On considering the evolution of standardized values of the five major capsaicinoids present in Malagueta pepper, it can be seen from Figure 4 that n-DHC, DHC and h-DHC (dihydrocapsaicin-like capsaicinoids) have a similar pattern of behavior whereas h-C and C (capsaicin-like capsaicinoids) show different evolution patterns.

The relative percentages of all capsaicinoids increase until the 33rd day of ripening and after this point they experience their first decrease in concentration. From this time, C and h-C show different behavior to the other capsaicinoids and they also differ from one another. h-C showed a marked decrease of 27.8%, whereas the decreases for DHC, n-DHC and h-DHC were less marked, corresponding to 5.1%, 4.7% and 2.5%, respectively. The concentration of C was practically constant at 79%.

With the exception of C and h-C, all capsaicinoids reached a minimum at 47 days of ripening, followed by an increase in concentration until the 61st day. Between days 61 and 68 there was a further drop in the relative concentrations of the capsaicinoids, once again with the exception of C. From this point (68 days→over-ripening of peppers) there was an increase in the relative percentage of capsaicinoids as the fruit dried out and the maximum concentration was reached for all capsaicinoids.

## 3. Materials and Methods

### 3.1. Chemicals

Capsaicinoid reference standards, i.e., capsaicin (97%) and dihydrocapsaicin (90%), were purchased from Sigma-Aldrich Chemical Co. (St. Louis, MO, USA). Water was obtained from a Milli-Q water deionization system (Millipore, Bedford, MA, USA). Glacial acetic acid and the methanol used for both the extraction of capsaicinoids and for the chromatographic separation were HPLC grade and were purchased from Merck (Darmstadt, Germany).

### 3.2. Pepper Crops

This study was conducted during the spring-summer season (April 2015–September 2015) in an automated greenhouse at the Agrifood Research Centre of Aragón (CITA-Zaragoza, Zaragoza, Spain). Average day/night greenhouse temperatures during the study period were 24/14 °C during spring and 27/19 °C during summer. Seeds were germinated in Petri dishes and, when cotyledons had developed, each plant was placed in a Jiffy-7 pot (Clause-Tezier Iberica, Almería, Spain). When the plants had three true leaves [six-week-old seedlings of Malagueta pepper cultivar (*Capsicum frutescens*)], each Jiffy pot was planted into a black plastic pot (one plant per pot; top diameter, 23 cm; bottom diameter, 17 cm; height, 18 cm). Plants were watered daily to maintain optimum growth.

### 3.3. Fertilization of the Plants

Plants were grown in a random distribution in a climatized greenhouse with a substrate mixture of peat, sand, clay-loam soil and Humin Substrat (Klasman-Deilmann, Geeste, Germany) (1:1:1:1, *v/v*). Two grams of Osmocote 16N-4P-9K slow-release fertilizer (The Scotts Miracle-Gro Co., Godalming, UK) were top-dressed on each pot at the beginning of growth.

### 3.4. Monitoring of the Ripening and Harvesting of Peppers

The evolution of individual and total contents of the five major capsaicinoids present in Malagueta peppers was studied. Peppers were marked at the end of flowering and hence the age of each pepper at the time of collection was known. Plants began to flower in mid-July. From this date, the new peppers that grew were marked with a temporal spacing of 7 days. The peppers were collected in the last week of September (plant ≈ 6 months old) and, from this date, the plant stopped producing peppers and sampling was therefore discontinued.

### 3.5. Plant Material

The Malagueta pepper variety (*Capsicum frutescens*) was employed in this study. Peppers were selected from 20 pepper plants cultivated in a greenhouse. Samples of different ages were obtained from all of the plants. The total amounts of peppers collected were in the range 232–346 g for different ages in order to avoid any particular effects from individual pepper fruit, as reported previously in the literature [27]. The stems and seeds of the peppers were discarded prior to analysis. Pericarp and placenta were subsequently ground together in a conventional mill to obtain a completely homogeneous sample. Aliquots of this sample were used for subsequent analyses. Once the peppers had been milled, they were frozen at –32 °C until analysis.

### 3.6. Extraction Procedure

The extracts from the pepper samples were obtained using an ultrasound-assisted extraction technique according to our previously developed method [48]. Ultrasonic treatment was carried out using a UP200S sonifier (200 W, 24 kHz) (Hielscher Ultrasonics, Teltow, Germany), with the sample immersed in a water bath coupled to a temperature controller (Frigiterm-10, J.P. Selecta, S.A., Barcelona, Spain). The capsaicinoids were extracted in a process with the following parameters: extraction solvent: methanol; temperature: 50 °C; output amplitude was the nominal amplitude of the transducer: 100% (200 W); duty cycle: 0.5 s; solvent volume: 25 mL; extraction time: 10 min; amount of sample: 0.25 g. The extracts were filtered through a 0.22 µm nylon syringe filter (Membrane Solutions, Dallas, TX, USA) prior to chromatographic analysis.

### 3.7. UHPLC-Fluorescence Analysis

The separation and quantification of capsaicinoids were carried out on a UHPLC (ACQUITY UPLC H-Class, Waters, Milford, MA, USA) system equipped with an ACQUITY UPLC Quaternary Pump System, an ACQUITY UPLC Auto Sampler with temperature control adjusted to 15 °C, a column oven set at 50 °C for the chromatographic separation, an ACQUITY UPLC^®^ Photodiode Array (PDA) Detector and an ACQUITY UPLC^®^ Fluorescence (FLR) Detector. Empower 3 software (Waters) was used to control the equipment and for data acquisition. 

**Figure 5:**
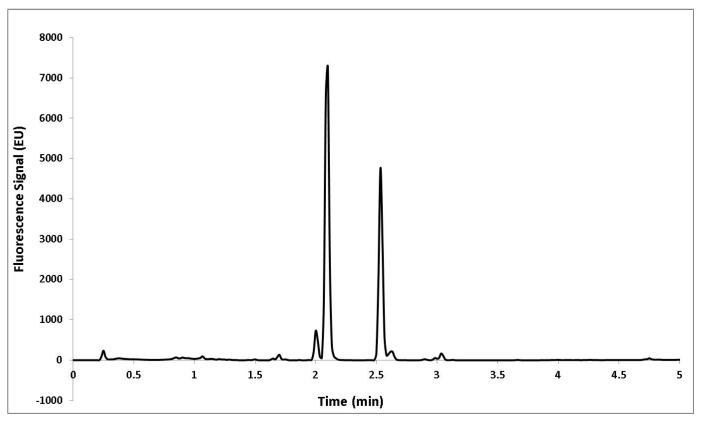
UHPLC chromatogram of Malagueta pepper extract. Fluorescence detection: excitation 280 nm; emission 305 nm. 1. Nordihydrocapsaicin; 2. Capsaicin; 3. Dihydrocapsaicin; 4. Homocapsaicin; 5. Homodihydrocapsaicin.

Capsaicinoids were analyzed on a Waters ACQUITY UPLC BEH C18 column (50 mm × 2.1 mm I.D., particle size 1.7 µm). A gradient method was employed for the chromatographic separation with acidified water (0.1% acetic acid, solvent A) and acidified acetonitrile (0.1% acetic acid, solvent B) working at a flow rate of 0.8 mL min^−1^. The gradient employed was as follows: 0 min, 0% B; 0.50 min, 45% B; 1.60 min, 45% B; 1.95 min, 50% B; 2.45 min, 55% B; 2.80 min, 63% B; 3.00 min, 63% B; 4.00 min, 100% B; 6.00 min, 100% B. The wavelengths employed for fluorescence detection were 280 nm (excitation) and 305 nm (emission). The injection volume was 3 μL. A typical chromatogram of Malagueta pepper is shown in Figure 5.

### 3.8. Identification of Capsaicinoids by Liquid Chromatography Coupled to Mass Spectrometry

**Figure 6:**
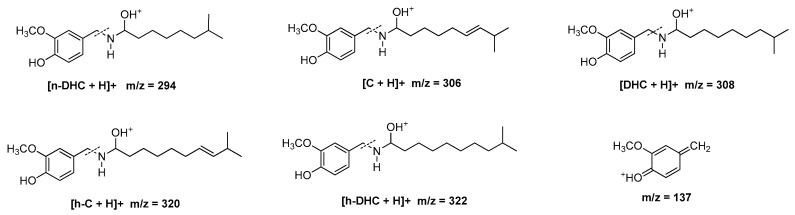
Proposed fragmentation pattern of protonated major capsaicinoids [M – H]^+^ with tentative structure for the product ion *m/z* = 137.

The five major capsaicinoids present in Malagueta peppers (n-DHC, C, DHC, h-C and h-DHC) were identified by ultra-performance liquid chromatography (UHPLC) coupled to quadrupole-time-of-flight mass spectrometer (Q-ToF-MS) (Synapt G2, Waters Corp., Milford, MA, USA). The injection volume was set to 3 μL. The chromatographic separation was performed on a reverse-phase C18 analytical column (Acquity UPLC BEH C18, Waters, 2.1 mm × 100 mm and 1.7 µm particle size). Masslynx software, version 4.1, was used to control the equipment and for the acquisition and treatment of data. The molecular ions [M + H]^+^ for the capsaicinoids identified had the following *m*/*z* ratios: nordihydrocapsaicin, 294; capsaicin, 306; dihydrocapsaicin, 308; homocapsaicin, 320; and homodihydrocapsaicin, 322. In the mass spectra of these five capsaicinoids (Figure 6), the *m*/*z* peak (137) that is characteristic of the fragmentation of capsaicinoids [49] was clearly observed.

The identification of capsaicinoids was achieved using water (0.1% formic acid) and methanol (0.1% formic acid) as mobile phases at a flow rate of 0.5 mL min^−1^. The elution gradient employed was as follows: 0 min, 0% B; 0.85 min, 55% B; 1.60 min, 55% B; 1.95 min, 60% B; 2.45 min, 63% B; 2.80 min, 70% B; 3.00 min, 70% B; 6.00 min, 100% B; 8.00 min, 100% B. The total run time was 12 min, including 4 min for re-equilibration. The determination of the analytes was carried out using an electrospray source operating in positive ionization mode under the following conditions: desolvation gas flow = 850 L h^−1^, desolvation temperature = 500 °C, cone gas flow = 10 L h^−1^, source temperature = 150 °C, capillary = 0.7 eV, cone voltage = 20 V and trap collision energy = 4 eV. Full-scan mode was used (*m*/*z* = 100–600).

### 3.9. UHPLC Calibration

The UHPLC method was used to obtain calibration curves for C and DHC (y = 1962318.42x + 64612.54 for C and y = 2147922.05x + 48033.91 for DHC), which are the two capsaicinoid standards that are commercially available. Regression equations and correlation coefficients (r^2^) were calculated using the ALAMIN software [50] (0.9997 for C and 0.9998 for DHC). The limits of detection (0.066 mg L^−1^ for C and 0.050 mg L^−1^ for DHC) and quantification (0.221 mg L^−1^ for C and 0.166 mg L^−1^ for DHC) were also calculated using the ALAMIN software.

### 3.10. Quantification of the Capsaicinoids Present in Malagueta Peppers

The five major capsaicinoids present in Malagueta peppers (n-DHC, C, DHC, h-C and h-DHC) were quantified. C and DHC were quantified from the calibration curves obtained from the standard solutions. Commercial standards of n-DHC, h-C and h-DHC are not available and these compounds were quantified from the calibration curve of DHC (for n-DHC and for h-DHC) and from the calibration curve of C (for h-C), given the structural similarities between these molecules. All analyses were carried out in triplicate.

## 4. Conclusions

Five capsaicinoids were identified in Malagueta peppers at different ripening times: n-DHC, C, DHC, h-C and h-DHC. An analysis of total capsaicinoids revealed a significant increase in the content of capsaicinoids in the early days of ripening (between 12 and 33). From this day on small ups and downs on the total concentration of capsaicinoids are observed. Therefore, from day 33 peppers could be collected if it is desired to harvest the peppers with a high capsaicinoid concentration. C was the major capsaicinoid, followed by DHC, n-DHC, h-C and h-DHC. A very slight variation was observed between the different percentages of individual capsaicinoids over the ripening period. Capsaicin and dihydrocapsaicin have very similar biological and spicy properties [46]. In this case it is verified that the two main capsaicinoids in the Malagueta pepper are the ones that present greater pungent and biological properties. On considering the evolution of standardized values of the capsaicinoids it was verified that n-DHC, DHC and h-DHC (dihydrocapsaicin-like capsaicinoids) show a similar pattern of behavior while h-C and C (capsaicin-like capsaicinoids) show different evolution patterns. The individual relationship of capsaicinoids can give an idea of the variety of pepper that has been used to make a spicy food. The time of harvest of Malagueta pepper with the aim of optimizing the total capsaicinoids content should be as late as possible. In any case, harvesting should be performed before over-ripening of the fruit is observed.

## Figures and Tables

**Table 1:** Malagueta pepper status at the time of harvest.

Code	Start of Fruit Development	Days Till Harvest	Visual State at Harvest
S-1	18/09	12	Green color
S-2	11/09	19	Green color
S-3	04/09	26	Green color
S-4	28/08	33	Green color
S-5	21/08	40	Green/red color
S-6	14/08	47	Red color
S-7	07/08	54	Red color
S-8	31/07	61	Red color
S-9	24/07	68	Red color
S-10	17/07	75	Over-ripeness

**Table 2:** Individual capsaicinoids (mg capsaicinoid g^−1^ FW) in Malagueta pepper during fruit ripening.

Day	n-DHC	C	DHC	h-C	h-DHC
12	0.035 ± 0.001	0.530 ± 0.021	0.229 ± 0.012	0.023 ± 0.001	0.013 ± 0.001
19	0.059 ± 0.002	0.761 ± 0.013	0.315 ± 0.017	0.033 ± 0.001	0.018 ± 0.001
26	0.078 ± 0.002	0.903 ± 0.043	0.430 ± 0.023	0.057 ± 0.002	0.025 ± 0.001
33	0.112 ± 0.006	1.268 ± 0.035	0.609 ± 0.022	0.086 ± 0.004	0.043 ± 0.002
40	0.105 ± 0.004	1.266 ± 0.065	0.574 ± 0.016	0.061 ± 0.003	0.041 ± 0.001
47	0.098 ± 0.003	1.350 ± 0.046	0.554 ± 0.019	0.084 ± 0.001	0.040 ± 0.002
54	0.104 ± 0.005	1.465 ± 0.021	0.615 ± 0.040	0.089 ± 0.003	0.048 ± 0.001
61	0.125 ± 0.006	1.384 ± 0.043	0.631 ± 0.032	0.085 ± 0.004	0.053 ± 0.002
68	0.117 ± 0.006	1.493 ± 0.054	0.630 ± 0.014	0.082 ± 0.004	0.048 ± 0.001
75	0.150 ± 0.009	1.589 ± 0.077	0.680 ± 0.030	0.090 ± 0.002	0.060 ± 0.005

Results are presented as mean ± SD, *n* = 3.
